# Supplementary material for: Chemical Solution Deposition of Barium Titanate Thin Films with Ethylene Glycol as Solvent for Barium Acetate
Source: Molecules. 2022 Jun 10;27(12):3753. doi: 10.3390/molecules27123753 (PMC9230800; doi:10.3390/molecules27123753)
Supplement: Supplementary file 1 [file molecules-27-03753-s001.zip › molecules-1726106-supplementary.pdf]

Supplementary Materials

Chemical Solution Deposition of Barium Titanate Thin Films with Ethylene Glycol as Solvent for Barium Acetate

Sabi William Konsago* [1,2], Katarina Žiberna [1,2], Brigita Kmet [1,2], Andreja Benčan [1,2], Hana Uršič [1,2], Barbara Malič[1,2]

1 Electronic Ceramics Department, Jožef Stefan Institute, Jamova Cesta 39, 1000 Ljubljana, Slovenia;
2 Jožef Stefan International Postgraduate School, Jamova Cesta 39, 1000 Ljubljana, Slovenia ;
* Correspondence: sabi.william.konsago@ijs.si, barbara.malic@ijs.si

Supplement S1. Thermal analysis of BT xerogels.

(a)-AcOH-MOE

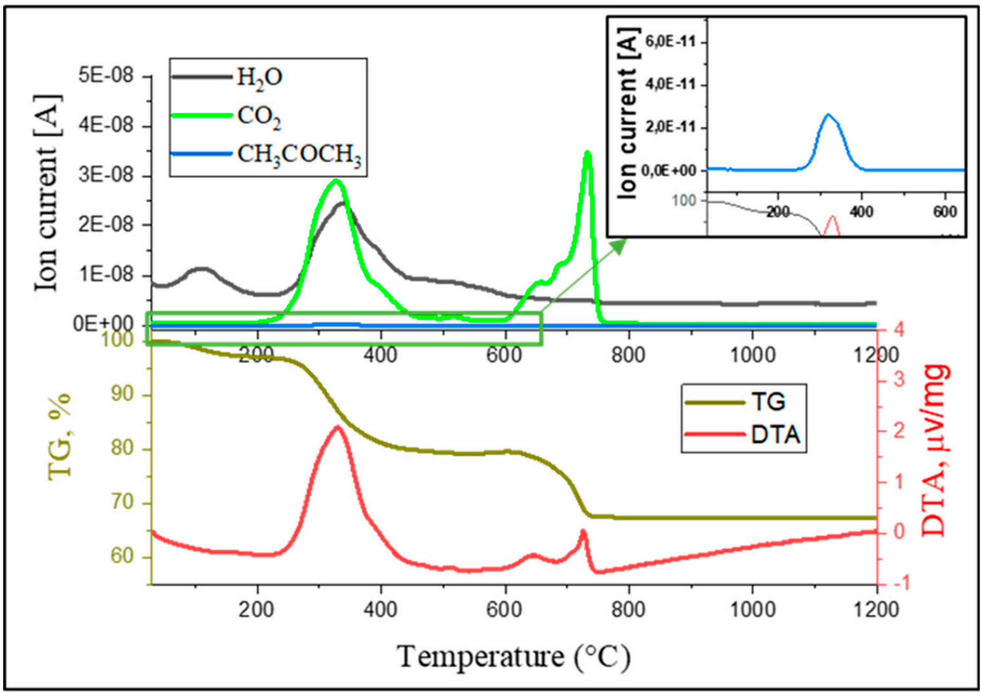

(b) EG-EtOH

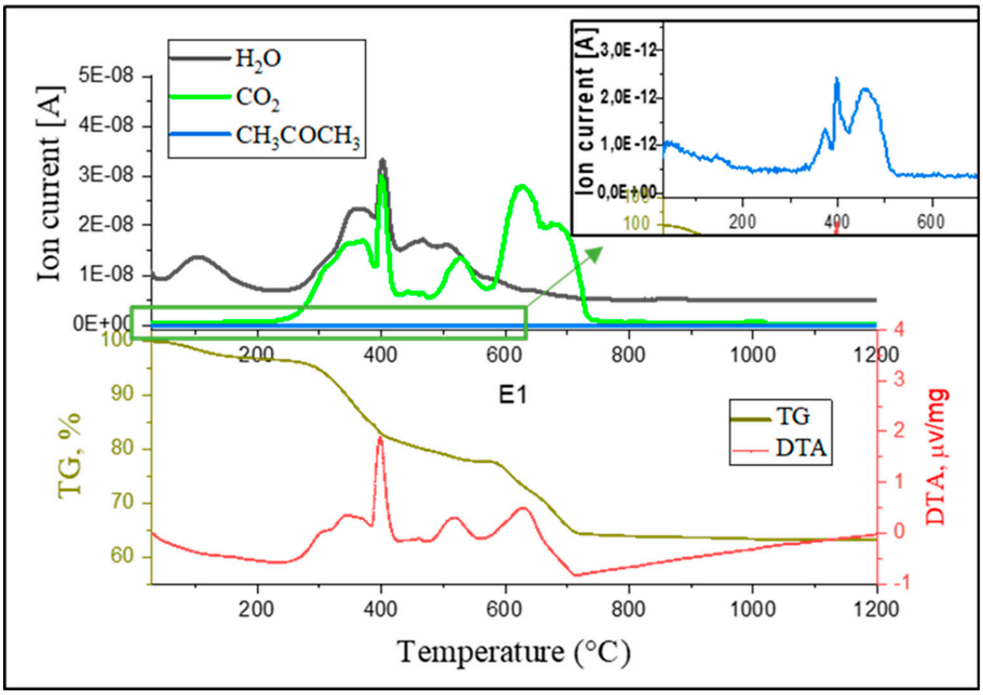

Figure S1. Thermal decomposition (TG, DTA) and EGA (H_2O, CH_3COCH_3, and CO_2) of (a)-ACOH-MOE, (b)-EG-EtOH xerogels.

(a) EG

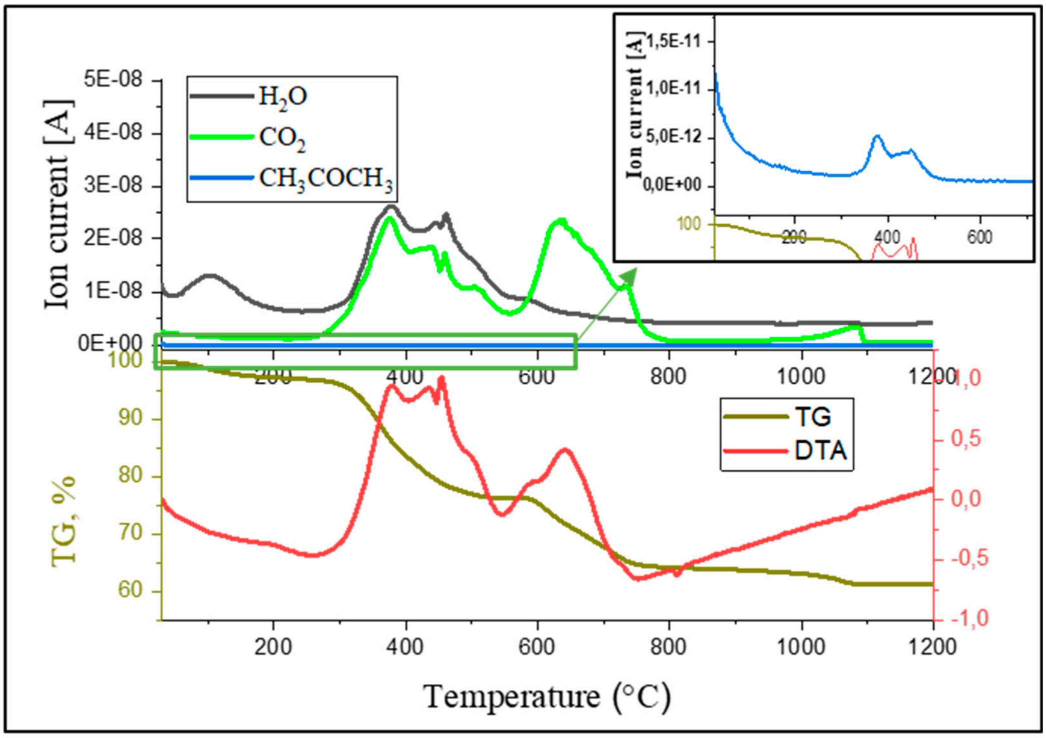

(b) EG-MOE

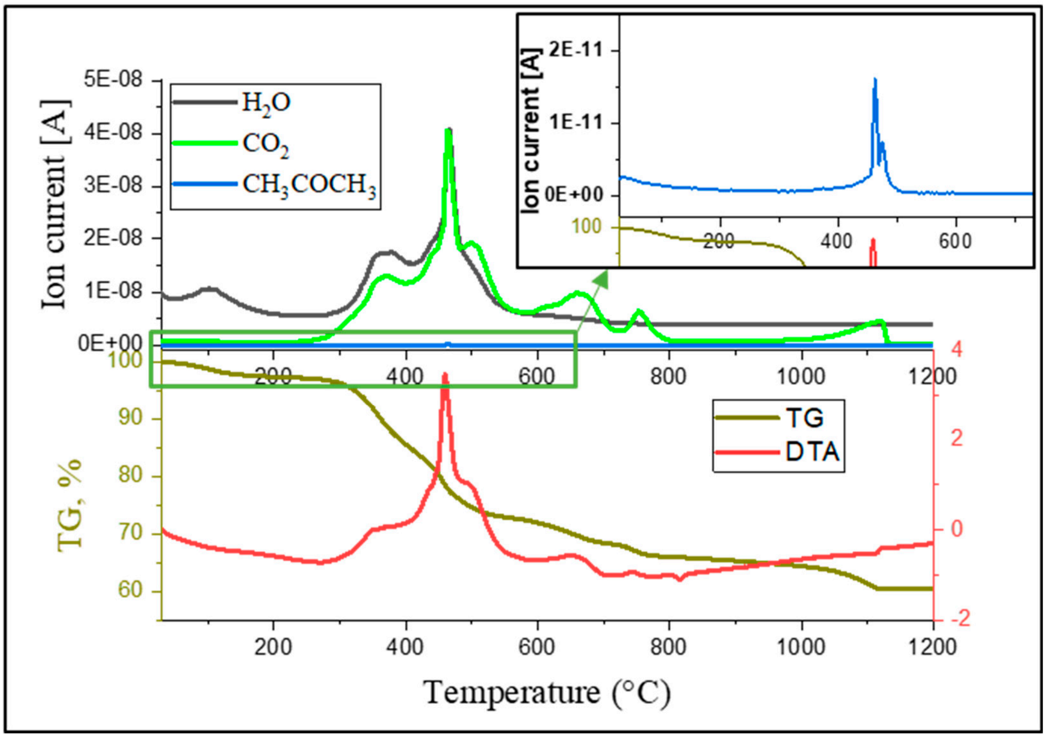

Figure S2. Thermal decomposition (TG, DTA) and EGA (H_2O, CH_3COCH_3, and CO_2) of (a)-EG and (b) EG-MOE xerogels.

Supplement S2. Crystallite size of $BaTiO_3$ (BT) thin films

In Table S1 crystallite sizes of BT thin films calculated using Scherrer equation (1) from the (110) reflection are collected. The films were prepared by drying at 250 °C, pyrolysis at 350 °C and rapid thermal annealing at at 800 °C.

Table S1. Crystallite size (d) of BT films

Film	FWHM, radian	d, nm
**2 minutes of drying and 2 minutes of pyrolysis processes**		
AcOH-MOE	0.00160	87
EG-EtOH	0.00234	59
EG	0.00264	53
EG-MOE	0.00147	94
15 minutes of drying and 15 minutes of pyrolysis processes		
AcOH-MOE	0.00152	91
EG-EtOH	0.00182	76

AcOH-MOE- BT film derived from the solution containing acetic acid and 2-methoxyethanol solvents,

EG-EtOH- BT film derived from the solution containing ethylene glycol and ethanol solvents,

EG- BT film derived from EG as the only solvent in BT coating solution,

EG-MOE- BT film derived from the solution containing ethylene glycol and 2-methoxyethanol solvents.

$$\mathrm{d} = \frac{K.\lambda}{L.cos\theta} \tag{1}$$

where d is the crystallite size, K is the Scherrer constant (0.9), λ is the wavelength of X-ray (0.15406 nm), L is the full width at half maximum (FWHM, radian) and θ is the peaks position of peak (radian).

Supplement S3. PFM analysis of BT films

Figure S3 shows the results of PFM analysis of $3 \times 3\ \mu m^2$ area scans of AcOH-MOE, EG-EtOH, EG, and EG-MOE BT films. In all cases, topography deflection images agree with SEM images regarding the grain size of the films. The PFM amplitude and phase images of all films show the local piezoelectric/ferroelectric responses.

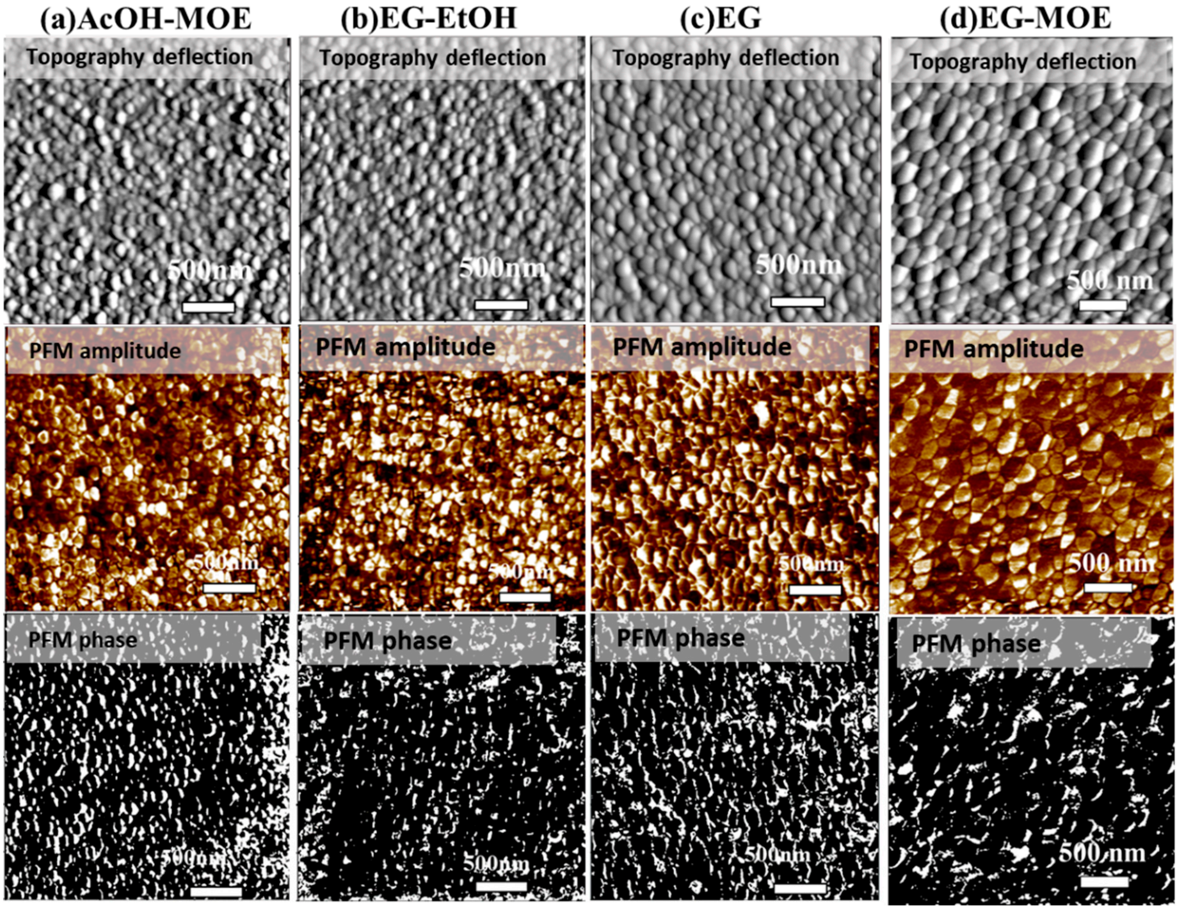

Figure S3. PFM analysis of BT films: Topography deflection, PFM amplitude, and phase images of (a) AcOH-MOE, (b) EG-EtOH, (c) EG, (d) EG-MOE films.

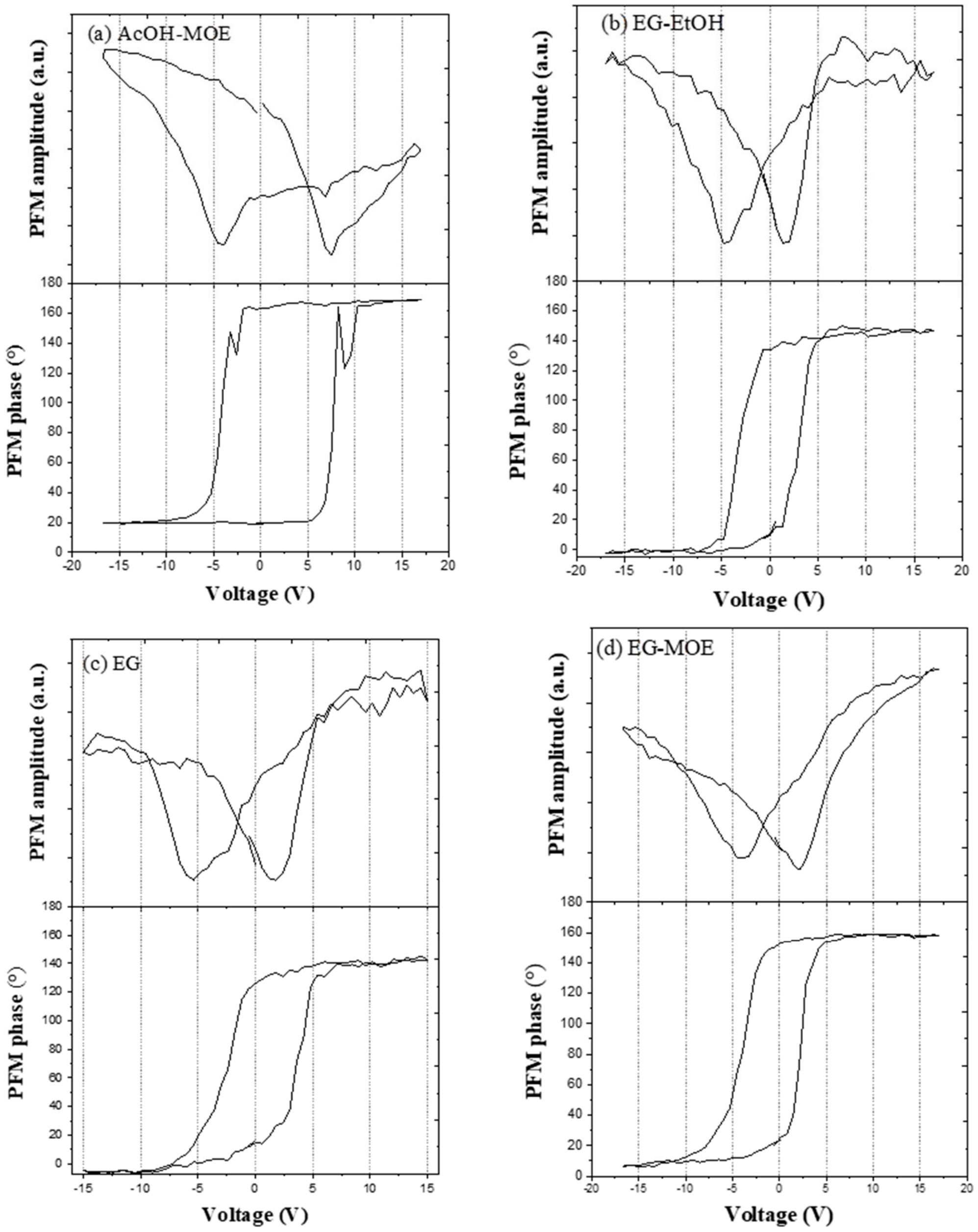

Figure S4. Local PFM amplitude and phase hysteresis loops of (a) AcOH-MOE, (b) EG-EtOH, (c) EG, (d) EG-MOE films.

Supplement S4. Conductive AFM (C-AFM)

Figure S5 shows the results of C-AFM analysis of 3 × 3 μm^2 area scans of AcOH-MOE and EG-EtOH films. As shown by the noise signal in Figure S5, no local electric current was detected in either sample. Therefore, the local current flowing through the samples was below ~1 pA, which is the detection limit of the C-AFM technique in ORCA mode [35].

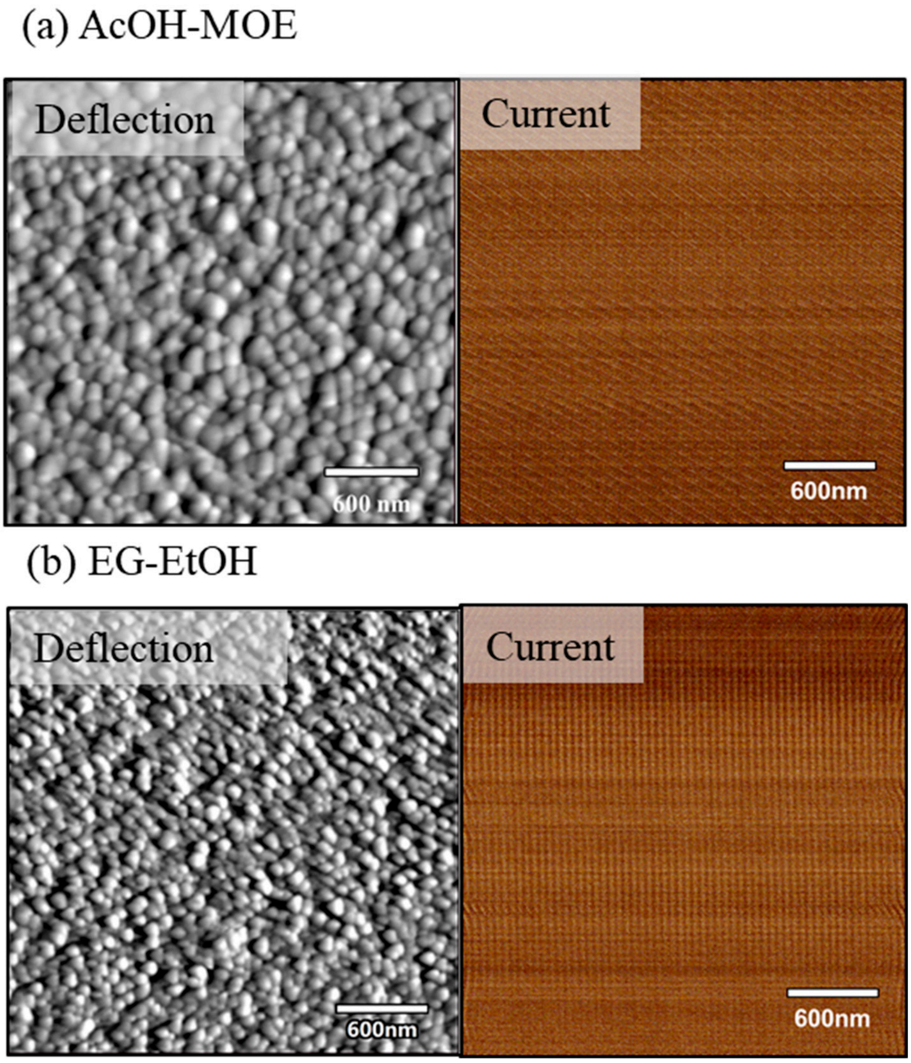

Figure S5. C-AFM analysis of (a) AcOH-MOE, (b) EG-EtOH films.

References

[35] ORCA - Conductive AFM, Asylum Research. Available at https://mundylab.umd.edu/wp-content/uploads/ORCASupportNote_rev1.pdf (accessed on 25 April 2022)
